# Bioactive Coatings with Antimicrobial Activity from *Pleurotus* spp. Biomass—A Literature Review

**DOI:** 10.3390/molecules31142481

**Published:** 2026-07-16

**Authors:** Joanna Zontek-Wilkowska, Przemysław Dorożyński, Bożena Muszyńska, Tomasz Skalski, Agata Krakowska

**Affiliations:** 1 Department of Inorganic Chemistry and Pharmaceutical Analytics, Faculty of Pharmacy, Doctoral School of Medical and Health Science, Jagiellonian University Medical College, 9 Medyczna Street, 30-688 Kraków, Poland; joanna.zontek-wilkowska@doctoral.uj.edu.pl; 2 Pharmlab Czarneccy Sp. z o.o., Lubostroń 15/6 Street, 30-383 Kraków, Poland; 3 Department of Inorganic Chemistry and Pharmaceutical Analytics, Faculty of Pharmacy, Jagiellonian University Medical College, 9 Medyczna Street, 30-688 Kraków, Poland; przemyslaw.dorozynski@uj.edu.pl; 4 Department of Medicinal Plant and Mushroom Biotechnology, Faculty of Pharmacy, Jagiellonian University Medical College, 9 Medyczna Street, 30-688 Kraków, Poland; bozena.muszynska@uj.edu.pl; 5 Biotechnology Centre, Silesian University of Technology, Krzywoustego 8 Street, 44-100 Gliwice, Poland; tomasz.skalski@polsl.pl; 6 Department of Analytical Chemistry and Biochemistry, Faculty of Materials Science and Ceramics, AGH University of Krakow, Al. Mickiewicza 30, 30-059 Kraków, Poland

**Keywords:** *Pleurotus* spp., bioactive layers functional coatings, antimicrobial properties, biofilms, hierarchical pattern layers, polysaccharides

## Abstract

*Pleurotus* is a genus of edible mushrooms widely cultivated worldwide on various substrates under controlled conditions. Its exceptional adaptability allows the production of biomass with increased macronutrient and mycochemical content. It is an excellent source of crude fiber and polysaccharides, particularly β-glucans, which are present in the dry mass of fruiting bodies, estimated as a percentage by weight (10.3% and 25.9%, respectively). The *Pleurotus* genus possesses a unique mycochemical profile, with numerous studies confirming its antibacterial, antiviral, anticancer, antioxidant, hypolipidemic, hypocholesterolemic, antihyperglycemic, and immunomodulatory activities. In the last 10 years, approximately 30 types of polysaccharides were isolated from *Pleurotus* spp., and their preliminary structures have been studied; however, their fine structures have rarely been reported. The polysaccharides showed immunomodulatory, hypoglycemic, anti-inflammatory, antioxidant, anti-aging, hepatoprotective, antitumor, and hypolipidemic activities, as well as regulation of intestinal flora, although the underlying mechanisms require further study. The unique potential of polysaccharides from *Pleurotus* spp. can be used as precursors of bioactive layers on various surfaces, including biomaterials, implants, medical devices and cosmetics. This paper reviews the state of the art and describes selected properties of polysaccharides derived from in vitro biomass from *Pleurotus* spp. cultures as a component of layer materials with confirmed antimicrobial activity. The mechanism of action of these polysaccharide layers on selected microorganisms is described. *Pleurotus* spp. are among the most valuable and common edible mushrooms species with polysaccharides being one of their active components. Their unique structures, biological activities and structure–activity relationships have been analyzed and discussed to explore future research directions and application prospects.

## 1. Introduction

Mushrooms are unique sources of nutritious components and beneficial active ingredients with confirmed pro-health value [1]. Whole fruiting bodies or submerged mycelium meet these new requirements and have been considered functional foods and significant sources of nutraceuticals. They can also be used as components of biomaterials [2], such as bioactive coatings and functional layers. Many species of mushrooms have been found to contain different bioactive components, such as polysaccharides, proteins and phenols which exhibit significant biological activities, such as anti-inflammatory, immunoregulatory, antidiabetic, anti-hyperlipidemic, hepatoprotective, antiviral and antioxidant activites [3,4,5]. *Pleurotus* spp. are the most advanced basidiomycete, widely distributed worldwide, and include more than 700 species and varieties. *Pleurotus* is one of the most precious and common edible mushrooms because of its easy cultivation, high yield and nutrient-rich content [1,6].

Oyster mushrooms were first described by Nicolas Joseph von Jacquin, an Austrian biologist, in 1775 as *Agaricus ostreatus*. The current scientific name, *Pleurotus ostreatus*, was assigned by the German mycologist Paul Kummer in 1871, who transferred it to the genus *Pleurotus* [1,2]. Since then, the genus *Pleurotus* has been described as the second most cultivated and distributed edible mushroom genus worldwide, after the champignon mushroom (*Agaricus bisporus*), because of its adaptability to different conditions [3,4,5]. This valuable species is characterized by unique properties, especially medical, biotechnological, and nutritional attributes, and its high content of fatty acids, steroids, and polysaccharides [6,7,8]. The exceptional properties of *Pleurotus* have attracted significant interest as a sustainable material for bioactive layers and functional materials [7,9], which could be used as a unique material for surface modification. Additionally, several *Pleurotus* species are highly adaptive, possess specific resistance to pests and polluted diseases, and do not require any specific conditions for their growth [8]. *Pleurotus ostreatus* grows throughout most part of Europe, including the United Kingdom and Iceland [8], and is widely distributed in many parts of Asia and North America [9]. This species is also an important member of the *Pleurotus* genus, which has significant medicinal importance and nutritional value [10] due to its antioxidative, anticarcinogenic, anti-inflammatory, anti-hypercholesteremic, antiviral, and immune-stimulating properties [10,11].

Different features of the *Pleurotus* genus are described with a special focus on their potential role in biomaterial engineering, medical devices, and surface modification. Bioactive substances with antimicrobial activity found in *Pleurotus* spp. biomass [12], which could be used as components of bioactive coatings, primarily derive from their secondary metabolites, such as terpenoids, polyacetylenes, phenolics, and the antibiotic 6-deoxyilludin, as well as specific compounds identified in *Pleurotus japonicus*. Additionally, glycoproteins such as indolone from *Pleurotus salmoneostramineus* filtrates have shown antimicrobial effects, particularly against bacteria such as *Pseudomonas aeruginosa* and *Candida parapsilosis*. The antimicrobial activity is generally effective against Gram-positive bacteria *Bacillus subtilis* and *Micrococcus luteus*, with some activity also noted against Gram-negative bacteria, depending on the *Pleurotus* species and cultivation substrate. Methanolic extracts from *Pleurotus ostreatus* and *Pleurotus florida* cultivated on agricultural waste have demonstrated significant antibacterial effects against pathogens, including *Escherichia coli*, *Pseudomonas aeruginosa*, and *Streptococcus faecalis*.

Furthermore, extracts from *Pleurotus* spp. exhibit additional bioactivities such as antioxidant, immunomodulatory, antiproliferative, and apoptosis-inducing effects, which enhance their potential as multifunctional bioactive agents in coatings for biomaterials or medical devices. Polysaccharides derived from *Pleurotus* mushrooms, a widely studied genus of edible and medicinal mushrooms, form bioactive layers with significant biomedical potential owing to their diverse biological activities and structural properties.

Earlier reviews on *Pleurotus* mainly summarized species diversity, extraction, chemistry, and biological activities, rather than a coating-focused translational framework, as presented in review papers from 2014 to 2021 [13]. Broader fungal-polysaccharide reviews focus on structure, extraction, immunomodulation, and health effects across many mushrooms; therefore, they are not specific to *Pleurotus*-to-coating translation. Bioactive coating reviews are application-driven but usually discuss chitosan, alginate, pectin, starch, proteins, essential oils, and plant extracts rather than *Pleurotus* polysaccharides specifically. Chitosan dominates this literature because of its confirmed antimicrobial activity, biocompatibility, and biodegradability. In comparison, reviews of coatings have focused on barrier function, controlled release, antioxidant and antimicrobial activity, and shelf-life extension of food products. Even more recent sustainability-focused reviews emphasize production strategies and material innovations rather than the selection of polysaccharides specific to *Pleurotus* species. Briefly, *Pleurotus* spp. are known to be rich in bioactive polysaccharides with antioxidant, immunomodulatory, antimicrobial, and functional properties.

The novelty of this review lies in its attempt to explain how bioactive coatings on the surfaces of medical devices or drug carriers can be developed based on conscious knowledge of the physicochemical properties of *Pleurotus* spp. polysaccharides. It is worth emphasizing that the current literature does not clearly synthesize the existing analyses.

## 2. Review Methodology

**Figure 1:**
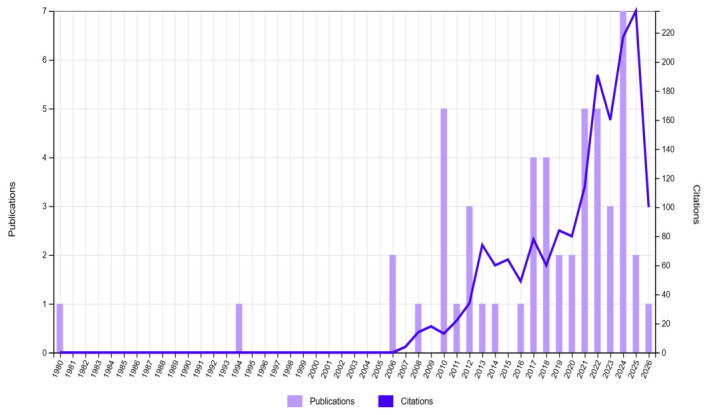
The citations report of papers published from 1980 to 2026 selected to further analysis (source: Web of Science).

A comprehensive literature search was executed across prominent scientific databases, including Scopus, Web of Science, PubMed, and Google Scholar, to identify pertinent studies concerning Pleurotus-derived polysaccharides and their applications in biomedical coatings and surfaces. The search strategy employed database-specific Boolean logic, integrating controlled vocabulary with open-ended keywords to locate relevant studies. The keyword domains encompassed *Pleurotus* spp., polysaccharides, β-glucans, pleuran, chitin, chitosan, functional coatings, bioactive coatings, films, and biomedical coatings. The search was confined to English-language publications and encompassed studies published from 1980 to 2026. The following study selection and eligibility criteria were applied in this review. Inclusion criteria: studies addressing the extraction, characterization, functional properties, or applications of *Pleurotus*-derived polysaccharides, with an emphasis on coatings, medical device coatings, implants, or other biomedical surface applications. Exclusion criteria included articles not directly relevant to the topic, those lacking sufficient methodological details, duplicates, conference abstracts, patents without experimental data, or articles lacking full text. Initial screening of titles and abstracts was conducted, followed by a full-text review of potentially relevant articles. The final selection of references was based on their relevance, methodological quality, and contribution to the review’s scope. The citations from the reviewed literature are depicted in Figure 1 (source: Web of Science), which outlines the study selection process and the references retained for analysis. Data extraction concentrated on core variables, including polysaccharide type (e.g., β-glucans, pleuran, chitin/chitosan derivatives), Pleurotus species, coating matrix or film type, reported antimicrobial or biological outcomes, type of evidence (direct Pleurotus-based data versus the broader context of the polysaccharide system), and limitations.

## 3. Polysaccharides

Polysaccharides produced by *Pleurotus* spp. can be classified into two main categories: exopolysaccharides (EPS) and intracellular polysaccharides (IPS). These compounds exhibit distinct features, production methods, functions, and bioactivities.

Exopolysaccharides are complex biopolymers secreted by fungal cells into the external environment. EPS is mainly located in fungal wall cells, creating boundaries that may facilitate interactions between the environment and other microorganisms. EPS plays a critical role in biofilm formation, cellular protection, adhesion to surfaces, and facilitation of nutrient acquisition [14]. EPS consists of diverse monosaccharides and typically has complex branched structures. Its composition varies depending on the fungal species and growth conditions [15]. Intercellular polysaccharides (IPSs) are stored within fungal cellsas energy reserves or cellular structural components. IPSs are often found in the fungal cytoplasm. The chemical structure of IPS is simpler than that of EPS, and it is primarily composed of polysaccharides, such as glycogen and storage glucans, that can be rapidly mobilized during periods of energy requirement or stress.

**Figure 2:**
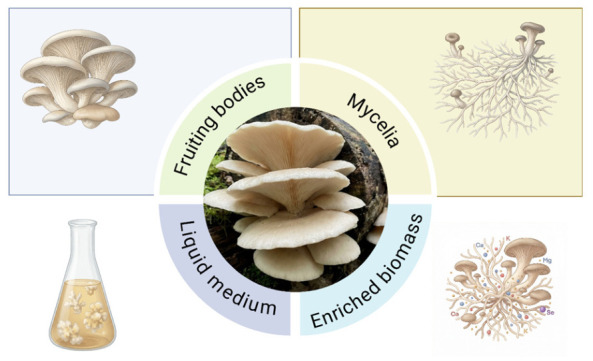
Schematic representation of fungal polysaccharide sources.

**Figure 3:**
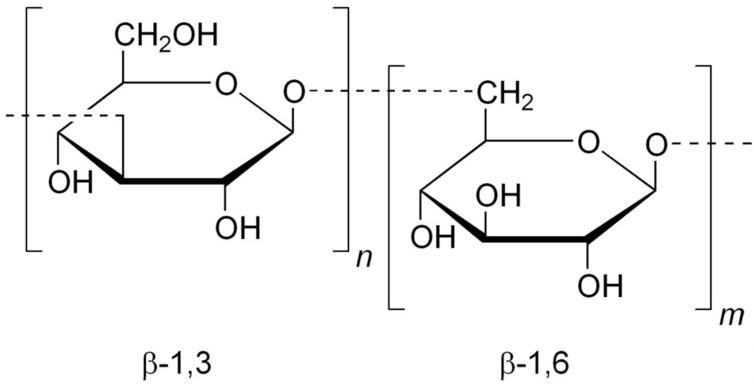
The formula for β- glucans β-1,3, β-1,6.

Fungal polysaccharides found in fruiting bodies and mycelia, referred to as condensation biopolymers, are primarily glucans, also known as glycans (condensation polymers) presented in Figure 2. The insolubility of glucan reaches 54–82% [14], and the solubility of β-glucan in water ranges from 16% to 46%, as confirmed by Gern et al. [15]. The main fungal polysaccharides are glucans with glycosidic bonds: (1 → 3), (1 → 6)-β-glucans, and (1 → 3)-α-glucans. Polysaccharides extracted from *Pleurotus* species, particularly *Pleurotus eryngii* and *Pleurotus ostreatus*, are typically rich in β-glucans, glucose polymers predominantly linked by β-1,3 and β-1,6 glycosidic bonds. These polysaccharides contain other monosaccharides, such as mannose and galactose, but are primarily glucose-based, with varying branching and molecular weights. The presence of β-glucan structures confers solubility, thermal stability, and bioactive properties to potential biomaterials and layers [16]. Figure 3 presents the formula for β-glucans.

### 3.1. β-Glucans in Pleurotus spp.

**Figure 4:**
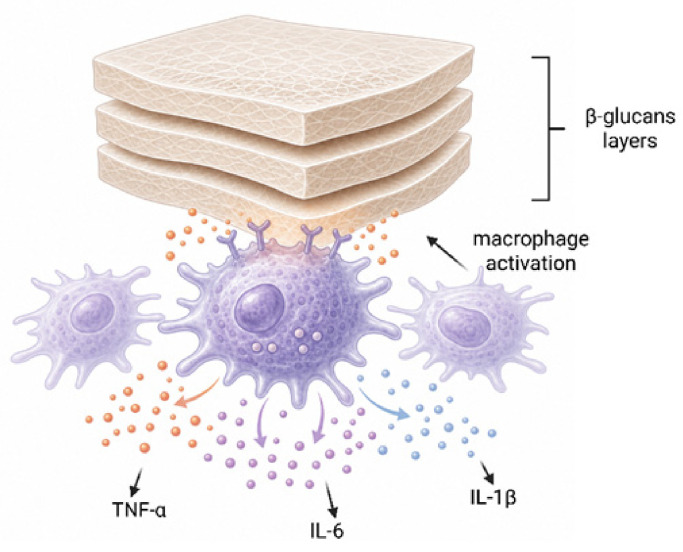
Schematic illustration of the activity of β-glucan layers.

β-glucans are well-known fungal polysaccharides that differ in structure and solubility in polar and non-polar solvents [17]. Soluble β-glucan fractions are characterized by their ability to dissolve in water and form viscous solutions. These molecules typically have a lower molecular weight and contain more β-(1 → 3) bonds, which are directly related to their solubility and bioactivity [18]. Polysaccharides present in the fruiting bodies and mycelia of *Pleurotus* spp. exhibit potent antioxidant activity by scavenging free radicals and chelating metals, thus protecting cells from oxidative stress. Vamanu [19] indicated that both intracellular and extracellular polysaccharides from *Pleurotus* exhibit potent antioxidant properties, as measured by assays such as DPPH scavenging and ABTS. Layers composed of β-glucans from in vitro cultures of *Pleurotus* can stimulate immune cells, such as macrophages, increasing cytokine production and modulating inflammatory responses within the implant [20]. The schematic action of the beta-glucan layer is presented in Figure 4 below. These phenomena are related to the β-glucan content and molecular weight, with purified high-molecular-weight β-glucan-rich fractions showing increased immunomodulatory capacities in various cell lines [19,20].

β-glucans are known to exhibit antitumor effects by activating host immune defenses and directly inhibiting tumor cell proliferation through biochemical pathways related to oxidative stress and immune modulation [21]. Novel extraction techniques such as subcritical water extraction using deep eutectic solvents, have been applied to isolate high-purity polysaccharide bioactive layers from *Pleurotus* spp., enhancing yield and preserving bioactivity [22]. Ion-exchange and size-exclusion chromatography are common purification steps for enriching β-glucan content.

**Figure 5:**
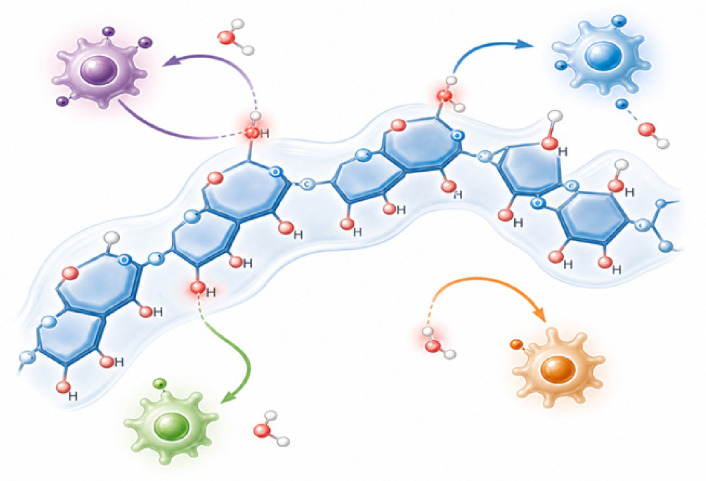
The activity of monosaccharide units.

Due to their biodegradability, low toxicity, and multifunctional bioactivities, *Pleurotus* polysaccharide layers are explored in wound healing, drug delivery, immune system modulation, and antioxidant supplementation. They are promising candidates for development of functional foods, nutraceuticals, and novel biomedical materials. β-glucan-based layers are known for their antioxidant, immunomodulatory, anti-inflammatory, and antitumor properties. Advances in extraction and purification have refined their biochemical characterization, revealing β-glucans as the principal bioactive components. These layers hold vast potential for therapeutic and industrial applications, owing to their natural biodegradability and biological efficacy [21,22]. Beta-glucans can scavenge various reactive oxygen species (ROS), including DPPH, hydroxyl, superoxide, and oxide radicals. This activity is due to the presence of hydrogen atoms in some monosaccharide units along their chains, which can be donated to neutralize free radicals [10,11,12,13,14,15,16,17,18,19,20,21,22]. The scavenging mechanism often involves hydrogen atom transfer (HAT) reactions in neutral polysaccharides and electron transfer (ET) reactions in acidic polysaccharides [23]. The schematic of the reactions is shown in Figure 5.

The antioxidant potential is closely related to the molecular weight, branching, and presence of specific sugar residues, such as β-glucans, which are prominent in *Pleurotus* species. β-glucans (with β-1,3 and β-1,6 linkages) are particularly effective at enhancing antioxidant defenses and can chelate transition metals such as Fe^2+^, preventing metal-catalyzed oxidative reactions and inhibiting lipid peroxidation, which protects cellular components from oxidative damage [23,24]. These polysaccharides can increase the activities of endogenous antioxidant enzymes, such as superoxide dismutase (SOD), catalase (CAT), and glutathione peroxidase (GPx), thus boosting the overall cellular antioxidant defense system. High-purity β-glucan-enriched polysaccharide fractions extracted from *Pleurotus eryngii* have demonstrated strong antioxidant activities, including reducing power and radical scavenging, making them effective in reducing oxidative stress [25].

### 3.2. α-Glucans

*Pleurotus* spp. contain both α- and β-glucans, with α-glucans constituting a smaller fraction relative to β-glucans in many samples. In a survey of several *Pleurotus* species, α-glucans were reported alongside β-glucans, with β-glucans typically dominating the glucan budget. α-glucans generally range from a few percent to around single-digit percentages, depending on the tissue and strain. The α-glucan content in *Pleurotus ostreatus* is estimated to be approximately 3–8% in caps and stems in some cultivars, with β-glucans often around 25–40% in similar samples, as confirmed by Synytsya A. et al. [26]. In a comparative study spanning 60 strains of *Pleurotus* spp., the α-glucan content was reported as a portion of total glucans, with values that were markedly lower compared to β-glucans, aligning with the general pattern described above by Golian et al. [26]. This species-wide assessment reinforces the view that α-glucans are present but are not the major structural glucan in *Pleurotus ostreatus* fruiting bodies (Golian et al., Synytsya A. [26,27]). α-glucans in *Pleurotus* (and other oyster mushrooms) have been described as linear or slightly branched α-(1 → 3)-D-glucans in several structural studies. Soluble α-glucans have been identified in alkali-soluble and water-soluble fractions, with some reports distinguishing linear α-(1 → 3)-d-glucan as an alkali-soluble component in oyster mushrooms, while β-glucans predominate as (1 → 3)-(1 → 6) glucans [20,21,22,23,24,25,26,27,28,29,30]. This structural framing is consistent with the general understanding that α-glucans are present in *Pleurotus* but are less abundant, and their isolation often requires specific fractions and conditions to capture them [29]. The presence of α-glucans has been explicitly discussed in reviews and primary reports focusing on glucan diversity in *Pleurotus ostreatus* and related species, noting that α-glucans can be a minority component but may contribute to the total glucan profile and be enriched under certain cultivation or extraction conditions [30]. The measurement of glucans in *Pleurotus* spp. typically uses Megazyme enzymatic assays to quantify total glucans and α-glucans, with β-glucans inferred by difference. This approach yields α-glucan fractions that are small relative to β-glucans in most *Pleurotus ostreatus* samples studied under standard conditions. However, the α-glucan content can be enhanced under particular cultivation substrates or processing, as suggested by substrate enrichment experiments in *Pleurotus eryngii* and related species, where the α-glucan content showed responsiveness to environmental factors [30,31,32,33].

### 3.3. Chitin and Chitosan

Chitin and its deacetylated derivative, chitosan, are structural components of the cell walls of *Pleurotus* spp. (oyster mushrooms). Across *Pleurotus ostreatus* and related species, chitin content is typically a minority portion of the cell wall polymers, often coexisting with abundant β-glucans and α-glucans in the cell wall. Chitin is present as α-chitin and/or in chitin–glucan complexes, and extraction methods (alkaline treatment, enzymatic steps) are able to liberate and characterize chitin and subsequently produce chitosan by deacetylation [26]. The reported chitin content in *Pleurotus* spp. tends be a few percent of the dry weight, although exact values vary by species, tissue (pileus vs. stipe), and extraction protocol [34,35,36]. Chitosan yields from *Pleurotus* species (mycelium and fruiting bodies) have been demonstrated in several basidiomycete studies, with mycelial chitosan obtainable under submerged or solid-state fermentation and fruiting-body chitosan documented for commercial mushrooms like *A. bisporus*, which was confirmed by Irbe et al. as a reference [34]. *Pleurotus* species possess chitin as a major cell wall polysaccharide alongside glucans, with the cell wall comprising a chitin–glucan–protein network. The chitin content in mushrooms generally ranges from low single-digit percentages to modest fractions of dry weight, depending on the species and tissue. *Pleurotus ostreatus* exhibits measurable chitin alongside glucans, but the chitin proportion is typically smaller than the glucan fractions [35,36,37,38,39]. Comparative studies have indicated that *Pleurotus ostreatus* stipes often contain higher glucan content than caps, while chitin content remains present in both tissues, enabling chitin isolation from different mushroom parts. Fractionation work has shown that chitin often coexists with glucans, forming CGC-like complexes in *Pleurotus* species [38].

#### 3.3.1. Structural Forms and Association with Glucans

Fungal chitin in *Pleurotus* is commonly found as α-chitin and as part of a chitin–glucan matrix. There is evidence for chitin embedded within a glucan-rich cell wall, with glucans (β-1,3 and β-1,6) intertwined with chitin [39]. This structural arrangement can influence both the isolation efficiency and the crystallinity of the extracted chitin, as well as the deacetylation behavior to form chitosan [40,41,42,43]. Studies focusing on mushroom chitin nanofibers have reported the retention of α-chitin crystallinity after the fractional removal of glucans, implying that mushroom chitin can preserve the α-chitin character even when glucans are present as surface-associated components [43].

#### 3.3.2. Chitin/Chitosan Extraction and Characterization from Pleurotus

Extraction methods typically begin with alkaline treatment to remove proteins and non-chitinous polysaccharides, followed by acidic purification steps to purify chitin from the remaining contaminants. Irbe et al.’s work [34] demonstrates that chitin derived from fungi can be extracted and then deacetylated, yielding chitosan with a characteristic nitrogen content, as confirmed by structural studies, including FTIR. *Pleurotus ostreatus* is a species widely studied for chitosan extraction, as evidenced by the reported chitosan yields from mycelia and fruiting bodies in various basidiomycete studies [34,35,36,37,38,39,40,41,42,43,44,45]. Currently, intensive work is underway to isolate chitin before converting it to chitosan, including from the stems of *Pleurotus* spp., which allows for more environmentally friendly processing and the production of higher-value by-products. Yin et al. [43] describe the isolation of β-glucans, lipids, polyphenols and proteins prior to chitin isolation, emphasizing that chitin derived from fungi tends to be less crystalline than chitin from crustaceans due to the remaining glucans, which may facilitate processing and further applications [46].

Chitosan production from *Pleurotus ostreatus* mycelium has been demonstrated in mycelial biomass studies [40,41,42,43,44,45,46,47], with chitosan yields varying by fermentation method (submerged vs. solid-state) and aeration. These studies show that *Pleurotus* mycelia can be a viable source of chitosan, providing alternative supply lines to crustacean-derived chitosan and enabling tailored material properties through controlled cultivation conditions [48,49]. Chitosan yields in *Pleurotus* fruiting bodies have also been reported in comparative surveys across Basidiomycota, where commercial mushrooms such as *A. bisporus* show notable chitosan content in the fruiting body, serving as a reference point for *Pleurotus* analyses [50,51,52,53,54]. This demonstrates that *Pleurotus* fruiting bodies can contribute to the chitosan supply, albeit typically at lower reported yields than crustacean sources and depending on species and processing [55,56,57,58,59].

FTIR, XRD, and elemental analyses are commonly used to characterize mushroom chitin and chitosan, with FTIR confirming characteristic amide bands and acetylation signals, and XRD providing crystallinity indices (CI) and allomorphic forms [60,61]. Studies comparing crustacean and fungal chitin have revealed differences in crystallinity and surface chemistry, which are influenced by glucan association. This highlights the need to adapt deacetylation and processing steps to fungal chitin when aiming for consistent chitosan products [60,61,62,63,64,65]. The broader literature emphasizes that chitin content is variable across Basidiomycota and even within *Pleurotus* spp., as it is influenced by extraction methods, thallus age, and tissue type [65,66,67]. Consequently, the reported chitin content of *Pleurotus* spp. should be interpreted within the context of the specific extraction protocol and tissue source [67,68,69].

**Figure 6:**
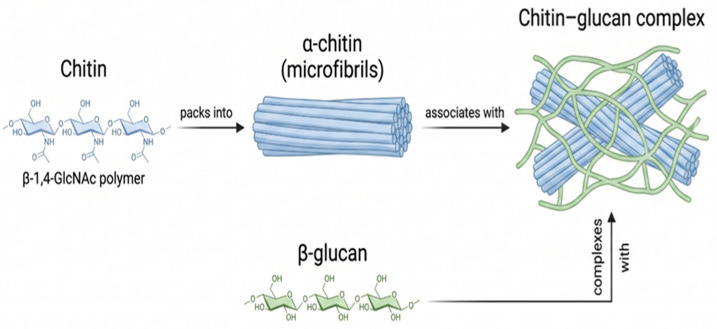
Schematic view of interaction between chitin and β-glucan complex.

Although *Pleurotus* chitin is clearly present and convertible to chitosan, there is variability in the reported chitin content and the proportion of chitin relative to glucans. Tupova et al. [66] emphasized the relatively low chitin content in *Pleurotus ostreatus* compared to glucans [67], while others demonstrated viable chitin yields from mycelium and fruiting bodies [68,69,70,71,72,73]. This reflects species- and method-dependent differences, including whether glucans remain associated with the chitin–glucans complex (CGC) after isolation, which can lower apparent chitin crystallinity and alter deacetylation outcomes [74,75,76,77,78,79]. A schematic representation of the interaction between chitin and beta-glucan complex is shown in Figure 6. The concept of chitin–glucan complexes in *Pleurotus* implies that the complete separation of chitin may require tailored enzymatic or chemical steps to minimize the loss of glucan-associated fractions that may be tightly integrated with chitin networks [80,81]. This nuance is highlighted in studies comparing fungal chitin isolation with crustacean analogs and in papers focusing on chitin nanofiber production from mushrooms [81,82,83].

### 3.4. Pleuran

Pleuran is a beta-(1,3)-D-glucan characteristic of *Pleurotus* species, particularly *Pleurotus ostreatus*, and is often discussed alongside other mushroom β-glucans. In *Pleurotus,* pleuran represents a major β-glucan fraction in many extracts and fractions; however the exact proportion relative to total glucans varies by tissue (fruiting body parts such as cap and stipe), extraction method (hot water, alkali), and species/strain [84]. The literature consistently identifies pleuran as a β-(1,3)-linked backbone with β-(1,6) branching in many preparations, and distinguishes it from other glucan fractions (e.g., β-(1,3)/(1,6) branched glucans and non-starch α-glucans) based on the Megazyme β-glucan assay and structural analyses [85,86,87,88,89]. Pleuran has been associated with immunomodulatory and potential health-promoting properties in mushroom-derived products, and its measurement is frequently reported in the assessment of total, α-, and β-glucans in *Pleurotus* spp. [89,90].

#### 3.4.1. Structural Forms of Pleuran and Association with Glucans

**Figure 7:**
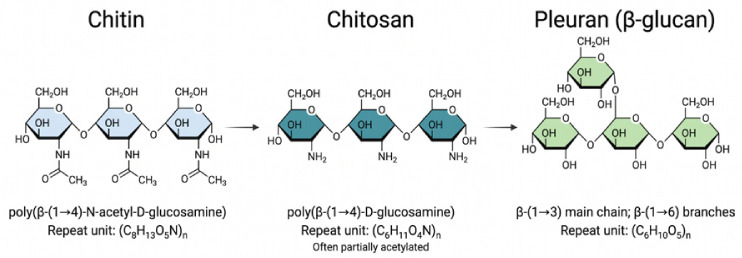
Comparison of the structures of chitin, chitosan, and pleuran.

Pleuran is a (1 → 3)-β-D-glucan found in *Pleurotus ostreatus* basidiocarps, often described as the main β-glucan component in oyster mushrooms. It is distinguished from other β-glucans by its linkage pattern (primarily β-1,3 with β-1,6 side chains) and by being part of the total glucan determination using Megazyme’s Mushroom and Yeast β-glucan kit [91]. Structural characterization across *Pleurotus* spp. indicates that pleuran coexists with other glucans (e.g., branched β-(1 → 3,1 → 6)-D-glucans) and can be associated with hot-water-soluble or alkali-soluble fractions, depending on the extraction protocol. The alkali-soluble fraction often contains linear α-(1 → 3)-D-glucans, whereas pleuran-type β-(1 → 3)/(1 → 6) glucans appear in hot-water-soluble fractions or specific alkali fractions, illustrating the complex glucan profile in *Pleurotus* spp. [91]. In *Pleurotus ostreatus*, total glucans comprise α- and β-glucans, with pleuran contributing to the β-glucan pool. Megazyme-based assays have revealed substantial β-glucan content in *Pleurotus* spp., with pleuran as a key constituent of the β-glucan fraction. The proportion of pleuran relative to total glucans is influenced by tissue (cap vs. stipe) and extraction method, aligning with broader observations that Pleurotus β-glucans are abundant, but PP varies across tissues and processing methods [91,92,93]. Comparative analyses across Pleurotus strains (60 strains, caps and stipes) showed that β-glucan content is a major contributor to total glucans, while α-glucans are comparatively smaller; pleuran-type β-glucan contributes to the measured β-glucan values, and its relative amount correlates with tissue type and genetic background [94]. Reports specifically naming pleuran as a principal β-glucan component in *Pleurotus ostreatus* are found in structural characterizations as presented in Figure 7; pleuran is charactered by -β(1 → 3) glucans in the main chain and β(1→6) glucans in the branches.

#### 3.4.2. Pleuran Extraction, Fractionation, and Analytical Considerations

Extraction methods (hot water, alkali) yield distinct glucan fractions, with hot-water-soluble fractions enriched in branched β-(1 → 3), (1 → 6) glucans and alkali-soluble fractions containing linear or branched glucans. Pleuran appears in several fractions depending on the conditions, and the Megazyme kit partitions glucans into α-, β-, and total glucans to allow calculation of the β-glucan fraction that includes pleuran-type polymers [89,90,91]. To quantify pleuran, researchers commonly use the Megazyme Mushroom and Yeast β-glucan kit, which separately quantifies total glucans, α-glucans (starch-like glucans after amyloglucosidase treatment), and β-glucans. This methodology implicitly categorizes pleuran as a part of the β-glucans. Structural studies support the presence of β-(1 → 3) backbones, characteristic of pleuran, in *Pleurotus* extracts [95]. Baeva et al. also reported near-complete structural profiling of *Pleurotus* glucans, noting that pleuran-type β-glucans can be solubilized under certain conditions and that glucan fractions may be co-isolated with other polysaccharides such as mannans or chitin complexes, which can influence the interpretation and yield of pleuran-rich fractions [95,96,97,98].

β-glucans from *Pleurotus* spp., including pleuran, are discussed in the context of their immunomodulatory activity and functional food applications. Reviews and primary studies emphasize the health-promoting potential of mushroom β-glucans, including pleuran, as part of the broader category of fungal β-glucans with potential immunostimulatory and anti-inflammatory effects [97,98,99]. This positions pleuran as a contributor to the health-promoting properties attributed to *Pleurotus* spp. products and mushroom-derived nutraceuticals, although specific in vivo or clinical evidence for pleuran alone remains an area of ongoing research [100]. In formulation contexts, *Pleurotus*-derived β-glucans, including pleuran, have been explored as functional ingredients in foods and dietary supplements, with attention to how processing affects glucan integrity and bioactivity. The bioactive potential and structural variability of mushroom β-glucans have been discussed in reviews that include pleuran among the major *Pleurotus* glucans, highlighting the need to standardize analytical methods for comparing pleuran across studies [101]. A key nuance is that “pleuran” is often used to denote the main β-(1 → 3) glucan fraction in *Pleurotus ostreatus*; however, glucan fractionation results can vary considerably depending on the extraction conditions and measurement methods. Megazyme-based measurements aggregate all β-glucans (including pleuran-type) into the β-glucan value; thus, unless structural fractionation is performed, pleuran is not usually reported as a separate numeric entity but as part of the β-glucan pool, as reported by Baeva et al. [102]. Some sources emphasize that *Pleurotus* β-glucans are predominantly pleuran-type β-(1 → 3)/(1 → 6) glucans, while others highlight complex glucan profiles with multiple β-glucan species; these descriptions reflect methodological differences in isolation, analysis, and reporting [103]. Consequently, cross-study comparisons require careful attention to tissue type, extraction method, and analytical protocol [104,105]. Differences in pleuran content among *Pleurotus* species (e.g., *P. ostreatus* vs. *P. eryngii* vs. *P. pulmonarius*) and among tissues (cap vs. stipe) further complicate direct comparisons, underscoring the need for standardized reporting in glucan profiling studies [102,103,104,105,106]. Pleuran is a distinct and biologically relevant component of *Pleurotus* β-glucans, typically categorized within the β-glucan pool defined by Megazyme assays. Its presence is well-supported in Pleurotus ostreatus and related species, with content varying by tissue type and extraction protocol. Structural studies have confirmed that pleuran-type β-(1 → 3) glucans are a major feature of *Pleurotus* glucans, often coexisting with other glucan forms in a glucan-rich cell wall matrix. Analytical approaches generally quantify β-glucans (and infer pleuran content) by difference from total glucans, making precise pleuran percentages dependent on the fractionation strategy used. The health-related discussions place pleuran within the broader context of mushroom β-glucans as potential immunomodulators and nutraceutical ingredients, though explicit pleuran-only activity data are less common and warrant further targeted study [99,100,101,102,103,104,105,106,107,108,109,110].

Pleuran use as a component of gel polysaccharides layers can modulate microbial adhesion and biofilm formation [110].

### 3.5. Chitin–Glucan Complexes

In *Pleurotus* species (oyster mushrooms), chitin commonly coexists with glucans as a major component of the fungal cell wall and is frequently organized as a chitin–glucan complex (CGC). This complex can take the form of CGC-like assemblies, where chitin is interwoven with β-glucans, influencing the extraction behavior, crystallinity, and downstream processing to chitosan or other value-added products [110]. Across *Pleurotus ostreatus* and related species, chitin content is typically a minority fraction relative to glucans; however its association with glucans (and its partial integration in CGCs) is a recurring theme in structural and analytical studies [111]. Extraction strategies (alkaline and enzymatic steps) can liberate chitin and enable its deacetylation to chitosan, with yields and crystallinity affected by the extent of glucan co-removal and CGC integrity [112]. Mycelial and fruiting-body sources provide viable chitosan, offering fungal-based alternatives to crustacean chitosan, with process conditions strongly influencing yield and polymer properties [113,114]. *Pleurotus* cell walls contain chitin alongside glucans (β-glucans and α-glucans) within a CGC framework. Chitin contents reported for *Pleurotus* spp. typically lie in a low-to-modest range of dry weight, and the chitin–glucan complex is a defining feature of mushroom cell walls that can persist after partial glucan removal, as confirmed by Irbe et al. and Grifoll et al. [34,42]. Tissue-dependent variation (cap vs. stipe) is noted in glucan analyses and has implications for chitin isolation since CGCs may be differentially distributed across tissues [115,116,117,118,119,120].

#### 3.5.1. Structural Forms of Chitin–Glucan Complex—CGC

Structural studies show chitin embedded within a glucan-rich matrix (Figure 6), with α-chitin observed in some contexts and β-glucans extensively intertwining with chitin in the CGC. This interpenetrating network can influence the crystallinity and the efficiency of deacetylation of chitosan during processing [120,121]. The primary structural motif involves a CGC, in which chitin (often α-chitin in some fungi) is interlaced with β-glucans (β-1,3 and β-1,6 linkages). This CGC arrangement is a characteristic feature of *Pleurotus* cell walls and contributes to the insoluble, recalcitrant portion of the wall, which is resistant to simple extraction. Structural characterizations identified fractions where linear α-(1 → 3) glucan or β-(1 → 3)/(1 → 6) glucans predominate in different soluble fractions, while insoluble residues retain CGC associations with chitin [121,122,123,124,125,126,127,128,129,130]. The crystallinity of chitin can be modified by the presence of CGCs, affecting deacetylation behavior and the properties of resultant chitosan [131].

#### 3.5.2. Chitin-Glucan Complex—Extraction, Fractionation, and Analytical Considerations

Extraction workflows to obtain chitin/chitosan from *Pleurotus* typically start with alkaline treatments to remove proteins and non-chitin polysaccharides, followed by acidic steps to purify chitin, and subsequent deacetylation to obtain chitosan. The extent of glucan presence and CGC integrity strongly influence both the yield and crystallinity of the recovered chitin and chitosan. Comparative analyses show mushroom chitin often exhibits lower crystallinity than crustacean chitin due to the presence of residual glucans and CGCs, which can be advantageous for processing and deacetylation into chitosan [132]. Fractionation strategies targeting by-products (e.g., β-glucans, proteins and lipids) before chitin isolation can improve greenness and economics, but may also leave CGCs partially intact in the chitin fraction, influencing downstream material properties. Grifoll et al. [42] demonstrated that CGCs can persist in mushroom-derived chitin and must be considered when interpreting chitin purity and crystallinity [133]. Analytical methods commonly employed include FTIR, XRD, elemental analysis, and NMR to assess the chitin content, degree of acetylation, crystallinity, and CGC-associated signatures. FTIR bands corresponding to amide groups reflect the presence of chitin, while residual glucans and CGCs modulate spectral features. XRD provides crystallinity indices that are influenced by CGC content [134].

*Pleurotus ostreatus* mycelium and fruiting bodies are demonstrated as sources of chitosan, with yields influenced by the cultivation mode (submerged vs. solid-state) and aeration. Mycelial chitosan production offers a scalable fungal-based route to chitosan, complementing fruiting-body-derived chitosan and enabling tailored material properties through cultivation conditions [135,136,137,138,139,140]. Reports have also documented chitosan yields in the fruiting bodies of *Pleurotus* and other basidiomycetes, noting the influence of species and processing on the final chitosan content [140]. Irbe et al. and Grifoll et al. underline that the chitin–glucan complex in *Pleurotus* can impact deacetylation efficiency and the resulting chitosan characteristics, including nitrogen content, molecular weight distribution, and solubility. These factors must be considered when selecting forage for industrial chitosan production or designing F-based biopolymer materials [141,142]. There is variability in the reported chitin content and CGC composition across *Pleurotus* species, tissues, and extraction methods. While chitin is consistently present, its proportion relative to glucans and the extent of CGC co-presence differ with tissue (caps vs. stipes) and species, complicating direct comparisons. This is attributed to methodological differences and biological variations, underscoring the need to specify tissue, species, and protocol when reporting chitin or CGC data [143].

Fungal chitin and chitin–glucan complexes (CGCs) from *Pleurotus* spp. are extracted using two broad strategies: (a) chemical methods (alkaline deproteinization followed by acid purification), and (b) enzymatic or greener approaches (protease, chitinase-assisted workups, ionic liquids, or deep eutectic solvents in some cases) to preserve native morphology and reduce environmental burden [144] CGC that is chitin embedded in a β-glucan–protein matrix, commonly influences extraction efficiency, crystallinity, and the deacetylation steps needed to obtain chitosan; method choice strongly affects final chitin and chitosan yields and properties [144,145,146,147,148,149,150]. Mycelial- and fruiting-body-derived chitosan have been demonstrated to be viable fungal sources, with yields and degrees of deacetylation (DD) modulated by cultivation mode (SF vs. SSF) and processing conditions [149]. Conventional chemical extraction (alkaline deproteination followed by acid treatment) is the most widely used method for *extracting* chitin/chitosan from *Pleurotus* and isolating CGC. This approach typically yields chitin with lower crystallinity than crustacean chitin because of residual glucans and CGCs; however it is scalable and well-characterized [148,149,150]. Reported chitin contents are modest (a few percent of dry weight) and heavily tissue- and species-dependent. Chitosan yields depend on deacetylation conditions and cultivation sources [150]. Greener or hybrid approaches aim to minimize harsh reagents, employing enzymatic deproteinization, milder alkali, or post-treatment fractionation to reduce chemical load while maintaining acceptable yields. Reviews and comparative studies emphasize that glucan association within CGCs can persist after alkali treatment, reducing the apparent chitin crystallinity and impacting subsequent deacetylation to chitosan [148,149,150,151,152,153,154,155,156,157,158]. Mycelial biomass-derived chitosan offers a scalable fungal alternative with yields that vary by strain and fermentation mode (SF vs. SSF; shaken SF typically yields higher biomass and SSF can maximize certain chitosan fractions). Fruiting-body chitosan remains viable but often lower in yield than mycelial sources. Extraction parameters critically shape DD and molecular weight, thereby affecting applications in biopolymers and layered systems [158]. *Pleurotus* chitin largely exists within chitin–glucan complexes, and extraction methods that effectively disrupt CGCs while preserving chitin integrity yield chitin with acceptable crystallinity for downstream deacetylation to chitosan. Green or enzymatic steps can reduce the chemical load but may alter the CGC disruption efficiency, influencing the final chitosan properties. Mycelial-based chitosan production provides scalable routes with process variables (SF vs. SSF, aeration) that tailor DD and MW [158,159,160]. Polysaccharides from *Pleurotus ostreatus* and *Pleurotus djamor* are mainly water-soluble β-glucans and heteropolysaccharides with strong antioxidant effects and other bioactivities. The choice of extraction method (water, enzymes, supercritical fluids, plasma, etc.) strongly affects the yield, structure, and functionality of extracted compounds.

The representative extraction conditions and outcomes (*P. Ostreatus*) are presented in Table 1.

## 4. *Pleurotus* spp. Polysaccharides as a Component of Bioactive Layers

Polysaccharides from *Pleurotus* spp. (pleuran, beta-glucans, chitin, chitosan and CGC) and other fungal polysaccharides have attracted interest as bioactive components of coatings which can be used on the surface of implants due to their antimicrobial, antibiofilm, immunomodulatory, and antioxidant properties. Peri-implant infections occur when aerobic and anaerobic bacteria colonize implant surfaces, evolving into resilient biofilms that defy host defenses and standard antibiotics [168]. Biofilms on dental or orthopedic implants compromise osseointegration, lead to inflammation, and may necessitate implant removal [169,170]. This problem is compounded by the limited success of systemic antibiotic prophylaxis alone and the need for surface-modified implants with durable antibacterial properties [171,172]. *Pleurotus* polysaccharides, as bioactive components of the layers, contribute to the improvement of the surface properties of the layer formation and bioactivity. A substantial corpus of work documents the antioxidant, antimicrobial, and antibiofilm activities associated with *Pleurotus* polysaccharides and crude extracts, providing a rationale for their inclusion in anti-infective surface layers [172]. These activities include DPPH and ABTS radical scavenging (antioxidant) and antimicrobial effects against a range of pathogens, often accompanied by other bioactivities, such as immunomodulation, which can contribute to host defense at the implant interface [172]. Although explicit transmission of these polysaccharides into multilayer coatings against peri-implant pathogens has not been uniformly demonstrated in the provided references, the intrinsic properties of *Pleurotus* polysaccharides, such as biocompatibility, gel-forming capability, and bioactivity make them attractive candidates for bioactive layers [173]. Non-biocidal antibiofilm strategies that leverage polysaccharide matrices can impede the initial adhesion of microbes and hinder biofilm maturation. Reviews of antibiofilm strategies highlight the potential of natural polymers, including mushroom-derived polysaccharides, to function as anti-adhesive layers or carriers for delivering antibiofilm agents. These concepts provide a mechanistic rationale for incorporating pleuran into interfacial coatings to reduce bacterial attachment and biofilm formation on the implant surface [168,169,170,171,172,173]. Antimicrobial and immunomodulatory actions: *Pleurotus* polysaccharides and extracts exhibit antimicrobial properties and can modulate host defense pathways, potentially enhancing the clearance of adherent microbes and reducing the infection risk at the implant interface [174]. In coatings, such bioactivities may function synergistically with other antibacterial components (e.g., metal nanoparticles and antimicrobial peptides) or provide biocompatible matrices that integrate with host tissues to support osseointegration while limiting infection. Layer-by-layer (LbL) assembly and other multilayer strategies enable precise control over the layer composition, thickness, and release kinetics. Polysaccharide layers can act as hydrophilic, biocompatible interfacial films that reduce protein adsorption and bacterial adhesion or as matrices for loading antibacterial agents. The literature supports the use of polysaccharide coatings on titanium and other implant substrates to modify interfacial properties, improve osseointegration, and confer antibacterial activity, with layered approaches offering tunable functionality. Layer-by-layer assemblies: LbL deposition of oppositely charged polymers enables the construction of multilayer films with pleuran as a constituent layer. Pleuran beta-glucan networks can contribute to hydrophilicity, biocompatibility, and potential interactions with microbial cell walls. LbL architectures can incorporate antibacterial polyelectrolytes, peptides, or inorganic nanoparticles to achieve dual action: reducing adhesion and delivering antimicrobial effectors when needed [175]. Hydrophilic hydrogel interlayers incorporating pleuran can act as moisture-retaining, biocompatible barriers at the implant interface, reducing nonspecific protein adsorption and providing a reservoir for bioactive agents. Such interlayers can be designed to release active compounds in response to inflammatory or microbial cues, aligning with infection prevention strategies in peri-implant care [176,177].

Pleuran, chitin, chitosan, and CGC can be integrated with inorganic antibacterial elements (e.g., silver, gold, zinc and copper) or bioactive ceramics (e.g., hydroxyapatite) to create composite coatings that promote osseointegration while presenting antibacterial interfaces. Reviews on titanium coatings and functionalized surfaces document the success of such hybrid approaches and the ongoing development of multilayered antibacterial strategies for implants [169,170,171,172,173,174,175,176,177,178]. Emerging research on mushroom-derived materials (and other biopolymers) suggests the potential of pleuran-containing scaffolds or printed layers with defined architectures that support tissue integration and localized antibacterial activity. Although most pleuran-layer literature focuses on coatings, the rheological properties of *Pleurotus* polysaccharides support their use in printable bio-inks and layered constructs for implant-adjacent applications [178,179]. Beta-glucan-rich networks from Pleurotus offer a biocompatible, hydrophilic matrix compatible with interfacial coatings and capable of forming gels or thin films, consistent with established principles for implant surface modification and biocompatible coatings [179,180]. These properties confirm that pleuran and other beta-glucans are valuable film-forming components in layered systems. Standardized reproducible polysaccharide-rich fractions with well-characterized molecular weights and branching are essential for coating applications. Purity, batch-to-batch consistency, and material stability under sterilization and physiological conditions are the key factors. Pleuran-containing layers should be biocompatible, avoid inducing adverse immune responses, and comply with biomedical device regulations [181,182]. Immunomodulatory effects can be beneficial but require careful evaluation to prevent undesirable inflammatory responses. The adhesion of the layers to titanium and other implant materials must be confirmed, and their compatibility with antimicrobial agents (e.g., metal nanoparticles, AMPs, and antibiotics) must be ensured. The protective and lubricating properties of polysaccharide layers should not affect osseointegration or mechanical stability. The peri-implant environment is subject to complex saliva/oil/tissue interactions, mechanical stress, and inflammation. The coatings must maintain their antibacterial efficacy and mechanical integrity over time, which requires accelerated aging and in vivo testing [183].

Fungal-derived polysaccharides, especially *Pleurotus*’ beta-glucans, CGC, chitin, and chitosan, represent an attractive class of materials for bioactive layered coatings owing to their biocompatibility, film-forming ability, and antimicrobial/antibiofilm potential, as confirmed by studies [168]. Although the available literature lacks specific published *Pleurotus* layer systems with adhesion/biofilm endpoints, the convergence of layer design principles, antimicrobial layer strategies, and *Pleurotus* bioactivity provides a strong rationale for targeted development. Table 2 presents examples of biological activity of polysaccharides from *Pleurotus* spp.

Crude and polysaccharide extracts from *P. ostreatus* stimulated the growth of *Lactobacillus paracasei* and other probiotics, while cell-free supernatants from *Lactobacillus* grown on mushroom polysaccharides showed strong inhibition of *Listeria monocytogenes* (36.3 mm). P. ostreatus polysaccharides on their own can inhibit some bacteria, particularly Gram-negative species like *P. aeruginosa*, and can enhance antibacterial effects when incorporated into materials (e.g., chitosan films) or by supporting probiotic growth [184]. However, the most potent antimicrobial activities of *P. ostreatus* so far involve mixed extracts, where polysaccharides act alongside phenolics and other compounds.

### 4.1. The Interaction Between the Surface Enriched with Fungal Polysaccharides Components and Microorganism

**Figure 8:**
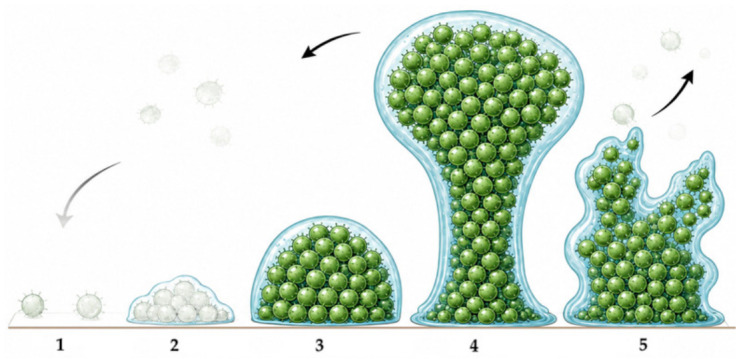
Diagram of bacterial biofilm formation. **1**—**Attachment**—bacterial adhesion to surfaces. **2**—**Microcolony**—bacteria attach to a surface, initiating the initial phase of growth and division, **3**—**Early biofilm**—irreversible connection. **4**—**Mature biofilm**—growth and maturation of bacterial colonies, the resulting bacteria form micro colonies around the point of attachment. **5**—**Dispersion**—release of microorganisms from the matrix and colonization of new surfaces.

The viability and proliferation of microorganisms on various materials, biomaterials, and implant surfaces depend largely on the physicochemical properties of the bioactive components present on the surface. Microbes often drift toward solid surfaces, leading to adsorption and subsequent colonization. The accumulation of bacteria, followed by microbial proliferation, is referred to as biofilm formation, as shown in Figure 8 [185].

**Figure 9:**
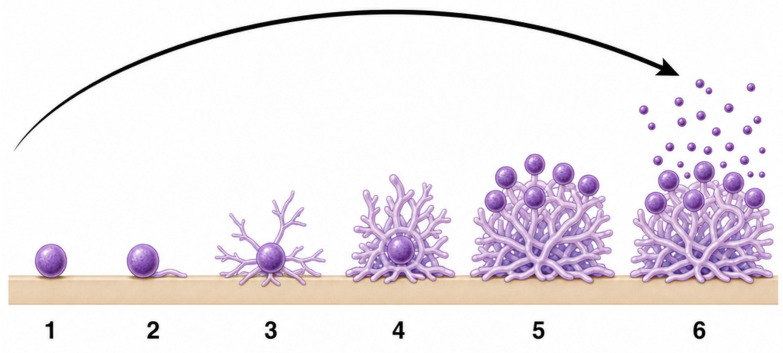
Diagram of fungal biofilm formation (**1**—**adsorption**, **2**—**fixation**, **3**—**microcolonies**, **4**—**early maturation**, **5**—**maturation**, **6**—**dispersion**).

During this period, microorganisms undergo various biological changes and create extracellular matrices (ECMs). The components of the ECM vary among microbes, and the species also depend on the growth conditions. In bacteria, the ECM primarily consists of polysaccharides, proteins, and extracellular DNA. These components serve various functions such as shielding free-living bacteria and assisting in genetic exchange. Furthermore, the ECM forms the foundation of biofilm, promoting interactions between cells and surfaces on nonliving materials [186]. In fungi, the extracellular matrix (ECM) provides resistance to antifungal agents, preventing them from reaching their intended targets on the surface or within fungal cells. Additionally, the ECM acts as a protective barrier against chemical and biological agents, playing a critical role in fungal biofilm formation (Figure 9) [187]. The charge on fungal cells, related to the presence of mannoproteins on the fungal cell wall, interacts with the surface of materials.

In the case of viruses, the extracellular matrix (ECM) is utilized for adhesion to target cells, followed by interaction with cell surface receptors, which allows viral entry [188,189,190]. As microbial cell attachment to surfaces is a crucial step in biofilm formation, understanding cell–surface interactions is important for controlling biofilm formation. Surface charge and wettability are key factors influencing microbial cell–surface interactions [189].

Bacteria are typically negatively charged. The cell wall of Gram-positive bacteria consists of peptidoglycan integrated with teichoic acids, which are anionic surface polymers that contribute to the negative charge of the bacterial cell. In Gram-negative bacteria, the outer membrane is composed of phospholipids and lipopolysaccharides, which confer a negative charge to the cell surface [168]. In molds and filamentous fungi, mannoproteins are linked to beta-glucans via glycosidic groups, further contributing to the negative charge of the fungal cell wall [189]. Negatively charged cells strongly interact with positively charged surfaces via electrostatic interactions. Furthermore, surface hydrophobicity, often resulting from adsorption, significantly influences adhesion and detachment from the surface, promoting biofilm formation. *Fimbriae* in bacteria are rich in hydrophobic amino acid residues, which are responsible for hydrophobicity and cell surface adhesion [190]. Various proteins are also closely related to cell surface hydrophobicity, influencing the adhesion of fungi such as *C. albicans* to surfaces. Microorganisms containing mycolic acid are more hydrophobic, and increasing the chain length of mycolic acid increases cell hydrophobicity [191]. Considering these factors, the development of antimicrobial surfaces is closely related to modifications in surface morphology and changes in pH. Polysaccharides from Pleurotus (oyster mushroom) influence microbial adhesion, biofilm formation, and their interactions with biological or material surfaces.

Polysaccharide extracts from *Pleurotus flabellatus* have been shown to significantly reduce the adhesion and biofilm formation of foodborne bacteria, achieving over 50% inhibition of *Enterococcus faecalis* growth. Clinical isolates demonstrated greater sensitivity than ATCC strains, indicating potential disruption of quorum-sensing mechanisms and initial surface attachment.

Antimicrobial polysaccharides: β-glucans derived from *P. ostreatus* exhibited potent antimicrobial activity, particularly against *Pseudomonas aeruginosa*, suggesting a direct inhibitory interaction with bacterial cells on the contact surfaces.

Exopolysaccharides from *P. pulmonarius* inhibited various microorganisms while promoting probiotic growth, indicating selective interactions with different microbes. This property is advantageous for Janus layer system applications.

Sulfated *Pleurotus* polysaccharides (from *P. eryngii* and *P. eous*) demonstrated enhanced antibacterial activity following chemical modification, resulting in increased inhibition zones for both Gram-positive and Gram-negative bacteria.

Chitosan combined with *P. ostreatus* polysaccharide films exhibited enhanced structural properties, including hydrogen bonding and Schiff base formation, and demonstrated significantly increased antibacterial activity against *E. coli* compared to chitosan alone [191,192,193,194]. This suggests that the incorporation of *Pleurotus* polysaccharides renders the surface more antagonistic to bacterial colonization. A gel-like extract of *P. ostreatus* stipe polysaccharides, comprising β-glucans and mannogalactans, formed viscous networks, was non-cytotoxic to Caco-2 cells, and facilitated wound closure in vivo. This indicates a biocompatible surface conducive to eukaryotic cell support, while potentially interacting differently with microbial entities.

Fermented *P. eryngii* polysaccharides interacted with intestinal mucus, dispersing polysaccharide aggregates and likely enhancing adhesion to the mucus layer, thereby influencing their retention and presentation to gut microbes. *P. eryngii* and *P. cornucopiae* polysaccharides underwent selective fermentation by the gut microbiota, resulting in an increase in beneficial taxa and short-chain fatty acids while suppressing opportunistic pathogens. This represents an indirect modulation of microbial colonization of the gut surface. In tadpole diets, *P. ostreatus* polysaccharides protected the skin and intestine from toxin-induced damage and normalized commensal microbial communities, thereby reducing Gram-negative, LPS-linked inflammation and enhancing resistance to pathogenic bacteria.

The antibacterial efficacy of polysaccharides is influenced by factors such as molecular weight, branching, monosaccharide composition, glycosidic linkages, charges, and functional groups. A lower molecular weight can enhance activity; for instance, a fungal polysaccharide with a molecular weight of 31.05 kDa exhibited stronger inhibition against *E. coli* and *S. aureus* than a 53.40 kDa polysaccharide, with minimum inhibitory concentrations (MICs) decreasing from 2.0 to 1.75 mg/mL for *E. coli* and from 1.2 to 0.85 mg/mL for *S. aureus* [191]. The activity is also concentration-dependent, as demonstrated by an Artemisia polysaccharide that exhibited dose-dependent inhibition of *S. aureus*, with an MIC of 1.25 mg/mL [193]. Charge-mediated interactions are crucial for antimicrobial action, as cationic polysaccharides bind to negatively charged microbial envelopes, altering membrane permeability. Functional groups elucidate the mechanism: amino groups are responsible for the antibacterial effects of chitosan, whereas sulfated or other ionized groups can sequester nutrients or interact with charged cell–surface targets. The structure and configuration also determine bioactivity, as branching, conformation, and non-carbohydrate substituents affect the solubility and biological behavior of polysaccharides derived from plants, microbes, and fungi.

### 4.2. The Mechanisms of Action of Pleurotus spp. Polysaccharides on Microbial Adhesion and Biofilms Formations Pleurotus

Polysaccharides engage microorganisms by directly diminishing surface adhesion and biofilm formation, thereby enhancing the antimicrobial efficacy of coatings, films, and gels. They also indirectly alter microbiota–mucus interactions in the intestinal and cutaneous environments. These attributes render *Pleurotus*-derived polysaccharides promising candidates for antimicrobial surfaces, functional foods, and protective biomaterials with barrier properties. *Pleurotus* polysaccharide preparations have been shown to inhibit microbial adhesion and biofilm formation in various laboratory models, particularly against *Enterococcus faecalis* and *Pseudomonas aeruginosa*. These polysaccharides primarily function as antiviral and anti-quorum sensing (QS) agents with low toxicity. However, current evidence is predominantly limited to in vitro studies and specific strains, leaving their broader antimicrobial and clinical significance to be fully established. *Pleurotus* (oyster mushroom) polysaccharides act as anti-adhesion and antibiofilm agents against bacteria by interfering with quorum sensing, early surface attachment, and biofilm matrix development.

In vitro studies conducted by J. Vunduk et al. [185] demonstrated that exopolysaccharide extracts from *Pleurotus* inhibited the adhesion and biofilm formation of foodborne pathogens, notably *Enterococcus faecalis*, with over 50% inhibition. These extracts likely interfere with quorum sensing (QS), which is interpreted as an anti-quorum sensing action that blocks intercellular communication, coordinating adhesion and biofilm formation. Crude polysaccharide extracts outperformed more purified fractions, suggesting that multiple polysaccharide components act synergistically, possibly through concerted effects on signaling and surface interactions. The polysaccharide extracts did not exhibit cytotoxicity against normal human cell lines, indicating a targeted effect on pathogenic bacteria rather than cell membrane disruption. Polysaccharide extracts from *Pleurotus ostreatus*, comprising the chitin–glucan complex, CGC, exhibit potent antibacterial activity against *Pseudomonas aeruginosa* by downregulating QS genes. This downregulation significantly reduced the mRNA levels of key QS synthase genes lasI, rhlI, and pqsA (by 2- to 17-fold), and virulence genes in *P. aeruginosa*. Fluorescence studies and SEM imaging revealed a significant reduction in the initial adhesion and biofilm formation. Low-molecular-weight compounds, such as vanillylacetone, in polysaccharide-rich extracts bind strongly to QS receptors (LasR, RhlR, and PqsR), acting as competitive inhibitors of autoinducers and likely altering receptor conformation.

**Figure 10:**
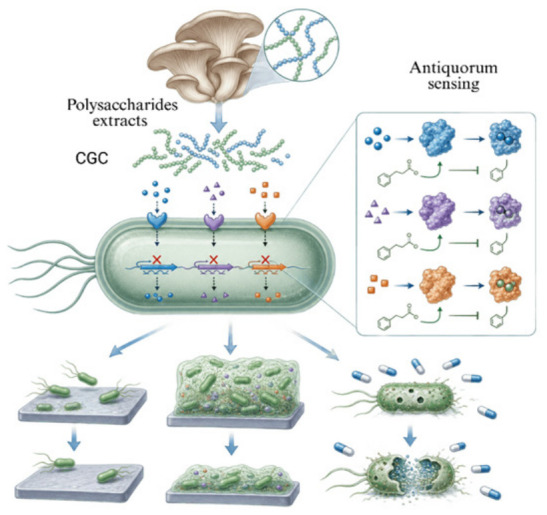
Mechanism of action of fungal polysaccharides against bacteria.

Fungal polysaccharides from *Pleurotus* increased antibiotic susceptibility by inhibiting QS and biofilms [188]. *Pleurotus* spp. polysaccharide extracts inhibit microbial adhesion and biofilm formation by disrupting the quorum sensing. This disruption involves the downregulation of QS genes and blocking of QS receptors, thereby suppressing early surface attachment, the development of an EPS-rich matrix, and virulence. These actions weaken biofilms and enhance bacterial susceptibility to antibiotics while remaining non-toxic to mammalian cells, as reported in previous studies. Figure 10 illustrates the mechanism of action of fungal polysaccharides against bacteria, encompassing biofilm stages such as initial adhesion to surfaces, matrix formation and maturation, virulence, and antibiotic tolerance.

Polysaccharides from *Pleurotus* species inhibit adhesion, quorum sensing (QS), and exopolysaccharide (EPS) production, primarily by interfering with QS signaling at the level of autoinducer synthase expression and receptor binding. This interference leads to the downregulation of virulence and EPS pathways and directly reduces initial surface adhesion, thereby limiting the development of mature EPS matrices. Strong evidence supports these effects in *Pseudomonas aeruginosa* and *Enterococcus faecalis* in vitro, although detailed gene-level EPS mechanisms remain only partially characterized in these species.

Although direct evidence is limited, substantial findings have been reported. Polysaccharides derived from *Pleurotus ostreatus* stems were combined with chitosan to form active composite films, with a 0.5% loading enhancing the tensile strength to 13,691 MPa. These composite films exhibited superior antioxidant and antibacterial activities compared to chitosan alone, including the inhibition of *Escherichia coli* growth [184]. Another study on a polysaccharide film from oyster mushrooms demonstrated that UV-C treatment increased tensile strength, decreased water vapor permeability, enhanced hydrophobicity, and smoothed the surface of the film. However, antimicrobial activity required the addition of leaf extract rather than relying solely on polysaccharide films. Materials derived from *Pleurotus* spp. have also been used as coatings. *Pleurotus eryngii* glucans served as reducing agents and encapsulated AgNPs, offering broad antimicrobial and antibiofilm activities with reduced tissue toxicity in vivo. β-glucans from *Pleurotus eryngii* were coated onto liposomes, and the study focused on the feasibility of coating, charge, molar mass dependence, and immune activation rather than surface antimicrobial activity [189].

Polysaccharide layers are primarily deposited on medical device surfaces using layer-by-layer (LbL) methods, dip coating, and their surface-preparation-assisted variants. These techniques are favored because of their mild aqueous conditions, film thickness control, and compatibility with natural polymers.

The layer-by-layer (LbL) method is the most extensively documented technique for fabricating polysaccharide antibiofilm and antifungal coatings on medical devices, such as catheters. This method involves the sequential adsorption of polycations and polyanions, typically in aqueous solutions with controlled pH and ionic strength, facilitating the construction of nanometric or sub-micrometer multilayers with predetermined compositions [189]. Systems incorporating chitosan (marine origin) or its derivatives as the cationic component and hyaluronic acid or alginate as the anionic component are frequently employed in medical applications. The LbL technique is particularly advantageous for medical devices because of its ability to apply conformal coatings on objects with complex geometries, including catheters and implants. Additionally, this method permits the incorporation of bioactive components between layers, enabling the coating to function as an anti-adhesive surface, contact fungicidal surface, or local drug reservoir [191]. The effective deposition of polysaccharide layers generally necessitates prior surface activation, which provides the functional groups and charge required for the stable adsorption of the first layer [190,191]. In studies involving PMMA, plasma-initiated grafting of poly(methacrylic acid) was employed, followed by the deposition of H-5/HA or TMC/SA multilayers, resulting in stable anti-Candida coatings [190]. Another approach involved creating a plasma-induced propanal interlayer with aldehyde anchor sites for the covalent immobilization of caspofungin, illustrating that interface chemistry is as crucial as the active substance. Substrate preparation can also enhance the hydrophilicity and uniformity of films. Alternative techniques, such as dip and spin coating, facilitate the deposition of smooth nanostructured polysaccharide layers [192,193,194,195].

## 5. Future Perspective

*Pleurotus* spp. biomass serves as a source of valuable active ingredients, notably polysaccharides, which exhibit significant biological activities and biocompatibility. These characteristics render them promising candidates for the development of bioactive coatings for medical devices or functional materials. Numerous in vitro and in vivo studies have substantiated their immunomodulatory, antioxidant, anti-inflammatory, anticancer, hypoglycemic, and antimicrobial effects, which are intricately linked to their structures and molecular weights [10,11,12,13,14,15,16,17,18,19,20,21,22,23,24,25,26,27,28,29,30,31,32,33,34,35,36,37,38,39,40,41,42,43,44,45,46,47,48,49,50]. Polysaccharide extracts, characterized by their strong gelling and film-forming properties, offer viscosity, stability, antioxidant and antibacterial activity, and promote epithelial cell growth, making them suitable for layers in direct contact with wounds. Exopolysaccharides from *P. pulmonarius* demonstrate exceptionally potent antimicrobial, antioxidant, and prebiotic properties. When incorporated into functional layers, they can function as drug transport surfaces while simultaneously providing prebiotic activities. Polysaccharide-based biomaterials are extensively used as surface coatings, hydrogels, and films because of their ECM-like structure, adjustable chemical composition, and favorable degradation properties. Currently, limited research and development on the structure–activity relationship (SAR) of fungal polysaccharides precludes a comprehensive explanation of the relationship between properties and the resulting structure. Further studies should incorporate structural analyses to ascertain these desired properties. Enriching polysaccharides through sulfurization, selenization, or the addition of bioelements such as magnesium or zinc can enhance the activity, stability, and antithrombotic properties of these coatings [190,191,192,193,194]. Polysaccharide coatings derived from *Pleurotus* encounter a translational gap between the bioactivity of the polysaccharides and the actual activity of the coating, necessitating further research. Detailed analyses of the coatings revealed that comparing antibacterial coatings remains challenging because of the absence of standardized antibacterial testing and the frequent underestimation of critical material properties, particularly for biopolymers. Research on *Pleurotus* indicates that the feasibility of coatings is contingent upon material characteristics, including molecular weight, structure, type of polysaccharide obtained, and the surface charge. Coating activity diminishes upon enzymatic degradation, with a minimum molecular weight of approximately 30 kDa. The use of in vitro cultures reduces variability, ensuring the consistent performance of polysaccharides derived from *Pleurotus* species.

The precise extraction and subsequent comprehensive structural analysis of fungal polysaccharides yielded a pure and consistently reproducible film-forming material. Polysaccharide biomaterials and films face several challenges, including batch variability, incomplete degradation studies, regulatory constraints, and the need to evaluate the effects of chemical modifications on their safety and functionality. The potential to precisely control macrophage polarization and immune response through polysaccharide coatings is promising; however, it requires extensive research. *Pleurotus* polysaccharides are known for their high bioactivity and compatibility. Initial studies on glucan-coated gels, films, exopolysaccharides, and liposomes have demonstrated the formation of functional bioactive layers with favorable properties. Future research should prioritize enhanced structural control, strategic chemical modifications, scalable extraction processes, and thorough in vitro and in vivo evaluations of biomaterial surfaces. The diverse biological activities of *Pleurotus* polysaccharides suggest their potential as bioactive coatings for medical and food-contact applications. Although direct studies on polysaccharide-coated hard medical implants have not been reported in these publications, several related studies indicate that this is a promising research avenue with significant application potential. There is a substantial demand for research on fungal polysaccharides and coating technologies, necessitating improved structure–function correlations, standardized antimicrobial assays, scalable production, and data-driven identification of design trends.

## Figures and Tables

**Table 1:** Comparison of extraction methods and outcomes of *Pleurotus ostreatus*.

Method of Extraction	Typical Conditions and Yield	Key Polysaccharides/Property	References
Conventional hot water	~70–100 °C, ~1 h; 5–11%	Antioxidant POP, β-glucans	[159]
MAE (aqueous)	Microwave heating	Highest soluble polysaccharides, phenolics	[159,160]
PAW-assisted water	PAW 700 W, 58 s; yield 11.67%	Higher antioxidant activity	[160,161,162,163,164,165,166]
Ethanol/salt ATPS	25% EtOH, K_2_HPO_4_	Simultaneous PS + protein, 90% recovery	[166,167,168]
Hot water supercritical CO_2_	25 MPa, 433 K	Antioxidant heteropolysaccharides	[168]
Subcritical water	180 °C; 20.35%	High yield, modifiable for anticoagulant use	[168]

**Table 2:** Antibacterial activity of polysaccharide-rich systems.

Form of Polysaccharides	Main Antibacterial Findings	References
Crude polysaccharide (phenol–sulfuric quantified)	Inhibits *B. subtilis*, *E. coli*; not *Candida*	[184]
Isolated β-glucans	Strong inhibition of *P. aeruginosa*, moderate of *S. aureus*	[168,169,170,171,172,173,174,175,176,177,178]
Chitosan–POP films (0.25–1% POP)	Composite films inhibit *E. coli* growth	[148]
Mushroom polysaccharides as prebiotics	Promote *Lactobacillus* growth; pathogen inhibition via probiotic supernatant	[158,159,160,161,162,163,164,165,166,167,168]

## Data Availability

No new data were created or analyzed in this study.
